# Editorial: Affecting, emoting, and feeling disability: entanglements at the intersection of disability studies and the sociology of emotions

**DOI:** 10.3389/fsoc.2025.1677289

**Published:** 2025-08-29

**Authors:** Yvonne Wechuli, Marie Sépulchre, Kelly Fritsch

**Affiliations:** ^1^Section Disability, Inclusion, and Social Participation, Department of Social Work, Faculty of Human Sciences, University of Kassel, Kassel, Germany; ^2^School of Social Work, Faculty of Social Sciences, Lund University, Lund, Sweden; ^3^Department of Sociology and Anthropology, Carleton University, Ottawa, ON, Canada

**Keywords:** affect, emotion, feeling, disability, disability studies, sociology of emotions, ableism

## 1 Introduction

This Research Topic engages disability as a vital yet underexplored domain within the Sociology of Emotions. It cultivates cross-disciplinary exchanges between the Sociology of Emotions and Disability Studies to deepen our understanding of emotions, feelings, and affect related to disability. Both fields conceptualize their core concerns as socially, culturally, politically, and ecologically situated. In doing so, they challenge dominant understandings that treat these phenomena as natural, ahistorical, or as confined to the realm of the human (Bericat, 2016; Thomas, 2007; Fritsch, 2022).

The emancipatory scholarship within Disability Studies—including subfields such as Mad Studies, Deaf Studies, and Critical Autism Studies—offers a rich repository of emotional and affective knowledge. Often grounded in first-person narratives and lived experiences, this scholarship uncovers affective and emotional dimensions of disability and challenges dominant paradigms within sociological thought. Disability Studies has engaged with questions of ontology, epistemology, performativity, and the more-than-human. Ontological inquiries into the nature of emotions, feelings and affect (Slaby and Mühlhoff, 2019) examine how disability and disabled emotions, feelings, and affect are shaped by experiences of, encounters with, and discourses about disability (Campbell, 2020; Hughes, 2012; Chen, 2012). Epistemological concerns focus on how emotions, feelings, and affect are known and understood (Flam and Kleres, 2015) in relation to disability as an experience, knowledge practice, and social category. Performativity-oriented research asks what emotions, feelings, and affects do (Ahmed, 2014; Wetherell, 2012). It highlights the affective and emotional toll of navigating dis/ableist structures and the multifaceted ways dis/ableism manifests through affective and emotional registers (Burch, 2021). This research embeds emotions, feelings, and affects around disability within broader social, cultural, and political processes by foregrounding their material and relational impacts (Thomas, 2007). Critical approaches to the more-than-human attend to how emotions, feelings, and affects produce, maintain, alter, or dismantle notions of disability. These perspectives have implications for the survival and thriving of disabled people, disability justice, and engagements with the more-than-human (Ray, 2017; Nocella, 2017; Clare, 2017).

## 2 Contributions to the Research Topic

A major contribution of this Research Topic revolves around the practices and impacts of disrupting affective and emotional expectations of disability. Many of the articles question taken-for-granted feeling rules (Hochschild, (2012) [1983]) and processes of affecting and being affected regarding disability and call attention to feelings and experiences that remain otherwise invisible. Frankel and Stern unpack how unpleasant affective states like anger are cast as alien affects (Ahmed, 2010) in solid-organ transplantation where patients are expected to show gratitude. Lafleur focuses on affective encounters between people and bodily remains on display in a museum. She offers alternatives to the museum's narrative frames by drawing on “the patients' perspective” and her own situatedness. Hiskies discusses how disability disrupts generic modes of responsivity to being affected, theorizing how impairment brings new affordances into the actionable and highlighting the socio-cultural negotiation of the body and the environment. Exploring the subjectification of parents of children with disabilities as “special parents,” Tröndle scrutinizes the gendered and ableist aspects of constructing the mother as the one who “suffers” from the situation. Bylund calls attention to the feelings provoked by austerity and the fear, disorientation, and insecurity, experienced by disabled people in the context of cuts to the Swedish welfare state. Finally, Taş questions the assumption that assistance dogs unconditionally love what they do by highlighting the affective labor they perform in interdependent human- animal-relationships.

Many articles in this Research Topic also contribute to the Sociology of Emotions through their adoption of relational approaches to disability and emotion. Karpicz et al. show the emotional labor that disabled archivists must perform to get access and accommodations in their workplace and note the feeling of ease and empowerment arising from collective approaches to access. In a different sort of workplace, Hultman and Hultman explore how it feels to live with personal assistance and perform emotion work at home. Moving into public spaces, Kubenz points to the emotional labor performed by disabled people who need to “walk on eggshells” in their everyday encounters with strangers who question their use of accessible parking spaces. Everyday encounters are also the topic of Ingram's study engaging with the impact that unsolicited advice by non-disabled people has on disabled people. Building on a relational approach, Hauser discusses how self-reflective emotion work embedded in social relationships can be performed in inclusive teacher education as a means to displace ableist practices. Finally, the polyphonic essay by Barden et al. explores whether a mixed-ability team of researchers working on learning disability history may be called an emotional community.

The 12 articles included in this Research Topic approach disability, affect, and emotions from different conceptual and methodological angles. Some of these contributions are theoretical (Taş; Hauser; Hiskes), others engage with diverse qualitative methods, including interviews (Bylund; Ingram; Karpicz et al.; Kubenz; Tröndle), ethnography (Frankel and Stern) or autoethnographic approaches (Barden et al.; Hultman and Hultman; Lafleur). These approaches resonate with Sauerborn and Albrecht's (2024) call for ethnographic, narrative, or autoethnographic methodologies as a way of capturing the observable, narratable, and experienceable aspects of affect. In addition to engaging Disability Studies and the Sociology of Emotions, the articles draw on various research fields, including Human-Animal Studies (Taş), History (Barden et al.), Museum and Curatorial Studies (Lafleur), Welfare State Studies (Bylund; Hultman and Hultman), and Human Geography (Kubenz).

## 3 Limitations of the Research Topic and publishing venue

What all contributions do have in common is to explore intersections of Disability Studies and the Sociology of Emotions coming from a Disability Studies perspective rather than from an explicitly Sociology of Emotions orientation. While disappointing, this is unsurprising given that one of us is the first author to publish on disability issues within the Sociology of Emotions in *Frontiers* as well as *Emotions and Society* (Wechuli, 2022, 2023). We hope this issue sparks new approaches in the Sociology of Emotions and continued work within Disability Studies.

The contributions are also geographically limited in scope, situated as they are in North America and Europe, largely reflecting our own Disability Studies networks and positionality. While our original call for articles attracted abstract submissions from beyond these regions, many authors ultimately published elsewhere due to the high open access publishing fees charged by *Frontiers* and additional access barriers during the submission and peer review such as issues with the submission platform and AI validation tools that incorrectly rejected articles that we, as guest editors, wished to consider.

As editors, we encountered a range of emotions navigating the *Frontiers* platform, which imposes rigid deadlines and an intense pace of labor we hadn't anticipated when agreeing to guest edit this Research Topic. Our inboxes were flooded with over 900 emails—many automated deadline reminders we couldn't easily adjust or turn off. In the context of unpaid academic labor and other professional obligations—teaching, grading, managing projects, or working additional jobs outside academia—this relentless acceleration caused significant stress, frustration, and anger. These pressures led to the loss of both authors and reviewers who couldn't keep up or who were fed up. Within Disability Studies, “crip time” is often forwarded to challenge normative timelines and enable new temporal orientations (Kafer, 2013). Yet, as (Kafer 2021) also notes, living within these alternative temporalities can feel anything but liberatory. For us, this clash between crip time and the platform's rigid demands underscores the need to support publishing systems that can better foster more accessible and care-centered forms of scholarship. This highlights the ongoing importance of Disability Studies and the need to engage with disabled knowledge and experience to transform our social and material worlds—an urgency powerfully reflected in the contributions to this Research Topic.
